# Molecular Characterization and Identification of *Calnexin 1* As a Radiation Biomarker from *Tradescantia* BNL4430

**DOI:** 10.3390/plants9030387

**Published:** 2020-03-20

**Authors:** Hye-Jeong Ha, Saminathan Subburaj, Young-Sun Kim, Jin-Baek Kim, Si-Yong Kang, Geung-Joo Lee

**Affiliations:** 1 Department of Horticulture, Chungnam National University, Daejeon 34134, Korea; ripple0727@naver.com (H.-J.H.); sami_plantbio86@yahoo.co.in (S.S.); zeroline75@empas.com (Y.-S.K.); 2 Devision of Environmental Science, Daegu University, Gyungsan 38453, Korea; 3 Korea Atomic Energy Research Institute, Jeongeup, Jeonbuk 580-185, Korea; jbkim74@kaeri.re.kr (J.-B.K.); sykang@kaeri.re.kr (S.-Y.K.); 4 Department of Smart Agriculture Systems, Chungnam National University, Daejeon 34134, Korea

**Keywords:** *Tradescantia* BNL4430, *Calnexin1*, ER chaperon, Irradiation tolerance, Biomarker

## Abstract

Calnexin (CNX) is an integral membrane protein that functions as a chaperone in the endoplasmic reticulum for the correct folding of proteins under stress conditions, rendering organisms tolerant under adverse conditions. Studies have investigated the cytogenetic effects of gamma irradiation (Ɣ-IR) on plants, but information on the molecular response under Ɣ-IR remains limited. Previously, we constructed a cDNA library of an irradiation-sensitive bioindicator plant, *Tradescantia* BNL4430 (T-4430) under Ɣ-IR, in which the *Calnexin-1* gene was highly upregulated at 50 mGy treatment. *TrCNX1* encodes a 61.4 kDa protein with conserved signature motifs similar to already reported *CNX1*s. *TrCNX1* expression was evaluated by semiquantitative reverse transcriptase PCR and quantitative real-time PCR and was ubiquitously expressed in various tissues and highly upregulated in flower petals under 50 mGy Ɣ-IR stress. The protective function of *TrCNX1* was investigated by overexpression of TrCNX1 in an *Escherichia coli* BL21(DE3) heterologous system. Using plate assay, we showed that TrCNX1 increased the viability of *E*. *coli* transformants under both UV-B and Ɣ-IR compared with the control, demonstrating that *TrCNX1* functions under irradiation stress. *TrCNX1* may enhance irradiation stress tolerance in crops and act as a radio marker gene to monitor the effects of radiation.

## 1. Introduction

During their life cycle, plants are challenged with adverse environmental factors under natural habitats, such as drought, salinity, extreme temperatures, and toxic chemicals. These stresses usually affect the cellular architecture and function of plants. In addition, intense light such as ultraviolet radiation (UV) can also regulate the physiological growth and development of plants [1], and ionizing radiation (IR) such as gamma rays has been implicated in diverse biological functions in plants [2]. At a cellular level, gamma radiation can penetrate cells and interact with molecules, resulting in the generation of reactive oxygen species (ROS). These ROS eventually induce cell death via oxidative damage to the cell membranes [3]. The functional effects of IR on plants, including *Arabidopsis*, rice, wheat, maize, soybean, and pumpkin have previously been studied [4].

Exposure of plants to IR, from either natural or synthetic sources, has induced marked biological effects, such as an increased rate of chromosome aberrations [5], seed germination [6], and plant height [7], and changes in flower color [8]. Most studies have used relatively a high dose (HD) of IR (1–15 kGy), with exposure for several minutes to an hour. According to the linear no-threshold (LNT) concept, the dose–effect relationship following exposure to both low-dose (LD) and HD IR may impact living beings (ICRP, 1990). Similarly, animal and plant cells have presented nonlinear dose-response curves resulting from the hypersensitivity of cells to low-dose IR (LDIR) (~100–50,000 mGy), and these cytogenetic responses could be difficult or unpredictable to high-dose IR (HDIR) [9]. These findings suggest that LDIR has more genetic consequences than HDIR. Epigenetic silencing or activation of genes through DNA methylation is the main molecular mechanisms underlying the response of eukaryotic cells to HDIR stress [10,11]. Although the cytogenetic effects of LDIR in human and nonhuman species have been previously reported, their responses towards IR at the molecular level remain unclear [9,12,13]; therefore, it is difficult to evaluate and measure the potential risks of LDIR on living organisms. Studies have demonstrated that plants may be used as bioindicators to quantify the biological effects of harmful environmental pollutants, such as heavy metals, xenobiotic chemicals, and radiation exposure [14].

Regarding the use of plants as bioindicators, T-4430 has been used to investigate the mutagenicity of different pollutants. Furthermore, it has emerged as a model plant for use in in situ monitoring systems to detect the effects of IR, which usually causes visible color changes in stamen-hair (STH) [15]. Recently, genes in rice showing global changes in their expression following exposure to different HDIR (40–200 Gy) have been identified as potential radio marker genes and have provided novel insights into the molecular radiation response of rice [16]. Hence, there has been increasing emphasis on the development of biomarkers using radio marker genes to pre-sense the environmental changes caused by natural or artificial radiation exposure under remediation. However, to date, there is no information on how plants respond to LDIR less than 1 Gy or 1000 mGy of IR. We previously aimed to identify putative novel radiation-responsive genes from transcriptomes (raw data were deposited at Gen Bank under the accession number PRJNA612745 (https://www.ncbi.nlm.nih.gov/bioproject/PRJNA612745) of T-4430 following exposure to LDIR (50, 100, 250, 500, and 1000 mGy). Through differentially expressed gene (DEG) analysis, we identified and isolated a putative novel *Calnexin-1* gene from *Tradescantia* (named *TrCNX-1*), which was abundantly expressed under 50 mGy (a fold change of 5.8), suggesting its potential role in the adaptive mechanism of *Tradescantia* in response to LDIR.

Calnexin (CNX) is a molecular chaperone protein located within the endoplasmic reticulum (ER) with a highly conserved structure and biological functions among plant, fungi, and animals [17,18]. CNX has been identified and characterized in multiple plant species, including *Arabidopsis* [17], maize [19], soybean [20], *Pisum sativum* [21], and *Oryza sativa* [22]. *CNX* genes have been implicated in stress tolerance in plants. Overexpression of a rice calnexin (*OsCNX*) in tobacco has been shown to confer tolerance against mannitol-induced drought stress [22].

Heterologous expression of plant cDNAs in *Escherichia coli* or yeast through functional screening assays is used to determine the specific function of genes of interest [23]. Functional screening of a cDNA library from a salt-tolerant plant, *Salicornia europaea,* in yeast resulted in the identification of a thaumatin-like gene, and overexpression of thaumatin-like proteins was found to confer salt tolerance to both yeast and *Arabidopsis* [24]. Similarly, a cDNA encoding the prolyl oligopeptidase gene from rice (*OsPOP5*) was overexpressed in *E. coli,* and OsPOP5 proteins were found to increase the tolerance of *E*. *coli* to various stress conditions, such as salinity, drought, and high temperatures [25]. Overproduction of Oshsp18.0-CII fusion proteins in *E. coli* increased the survival of *E. coli* cells in response to UV-B stress; the results further revealed that Oshsp18.0-CII provided thermotolerance [26]. Thus, functional screening in microorganisms may be used as a rapid and effective method to characterize specific genes derived from heterologous species based on their specific functions.

In the present study, we report the molecular and functional characterization of a putative novel radiation-responsive gene, *TrCNX-1,* in T-4430. TrCNX-1 was found to be closely related to CNXs from monocotyledons. Results from semiquantitative reverse transcriptase PCR (RT-PCR) and quantitative real-time PCR (RT-qPCR) investigations indicated that *TrCNX-1* was highly upregulated under ~50 mGy, suggesting that this gene may have a specific role in the adaptive functions of T-4430 in response to LDIR. Furthermore, overexpression of TrCNX-1 protein in *E*. *coli* demonstrated that this protein confers irradiation tolerance to *E*. *coli* under IR (Ɣ) and non-IR (UV-B) conditions.

## 2. Results

### 2.1. Identification and In Silico Analysis of Putative CNX1 Gene from *Tradescantia*

The assembled transcriptome data of *Tradescantia* clone BNL 4430 revealed that the *Tradescantia Calnexin-1* (*TrCNX1*) gene was hypersensitive to LD of Ɣ-IR (50 mGy). Based on the *TrCNX1* transcript sequences, we designed allele-specific PCR primers (Table S1) to amplify TrCNX1*-*encoding genes from the genomic DNA and cDNA of T-4430. 

**Figure 1:**
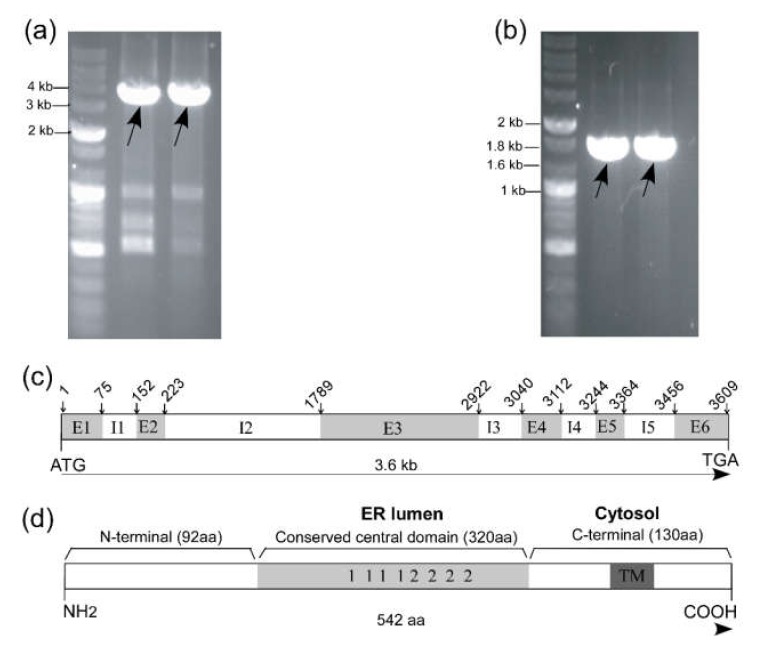
Isolation and identification of the *TrCNX1* gene in *Tradescantia*. (**a**) PCR amplification of *TrCNX1* from genomic DNA. (**b**) PCR amplification of *TrCNX1* ORF from cDNA of *Tradescantia*. Target-specific amplicons indicated by an arrow. (**c**) Structural analysis of the *TrCNX1* gene. In *TrCNX1*, the start (ATG) and stop (TGA) codons are shown in the gene coding region. The numbers above the *TrCNX1* gene represent the DNA base pair (bp). Corresponding positions of introns (I)/exons (E) are indicated by arrows with the bp position. Numbers of introns (I1–I5) and exons (E1–E6) are denoted by white and gray boxes, respectively. (**d**) General structural features of the TrCNX1 protein. The putative conserved domains are shown according to previous reports [27]. The gray box indicates the conserved central domain of the endoplasmic reticulum (ER) lumen and those numbered 1 and 2 represent Ca^2+^-binding regions (motif 1) and oligosaccharide-binding domains (motif 2), respectively. The dark gray box represents the transmembrane (TM) domain in the cytosol. The size of each domain is denoted in closed brackets with amino acid (aa) residue lengths.

Amplicons of 1.8 kb (Figure 1a) and 3.6 kb (Figure 1b) were amplified from cDNA and genomic DNA, respectively. After DNA sequencing, intron–exon analysis revealed that the *TrCNX1* genomic sequence comprised of six exons (E) and five introns (I) in its gene coding region (Figure 1b). NCBIs ORF finder analysis indicated that the cloned cDNA of *TrCNX1* was 1629 bp in size and encoded a protein of 542 amino acids (Figure 1d) with a predicted theoretical molecular mass of 61.4 kDa and a pI of 4.69. 

**Figure 2:**
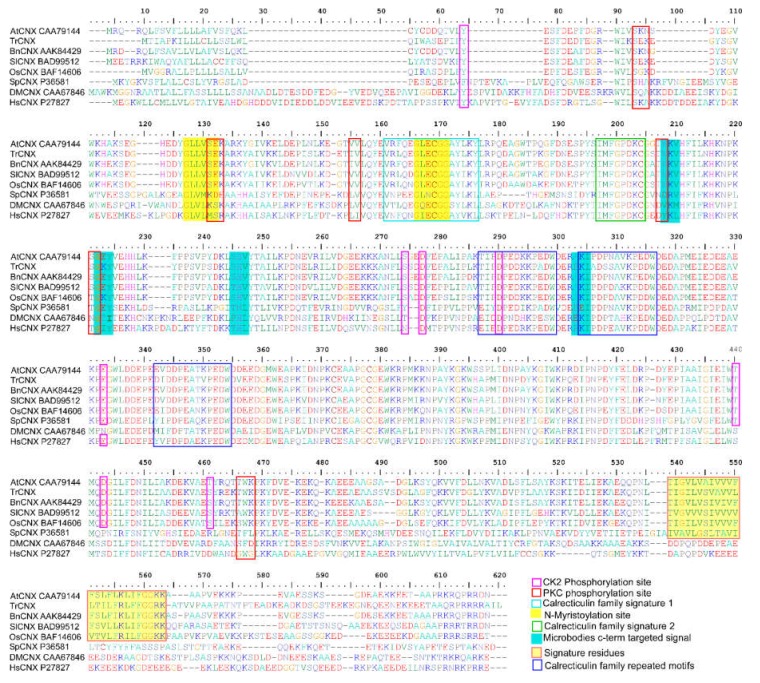
Comparison of deduced amino acid sequences of *calnexin* (*CNX*). Multiple sequence alignment of the TrCNX1 protein with various plant and non-plant CNX proteins. At, *Arabidopsis thaliana* (CAA79144); Tr, *Tradescantia* (KU530113); Br, *Brassica napus* (AAK84429); Sl, *Solanum lycopersicum* (BAD99512); Os, *Oryza sativa* (BAF14606); Sp, S*accharomyces pombe* (P36581); Dm, *Drosophila melanogaster* (AAF48618); Hs, *Homo sapiens* (P27824.2). Distinct structural features of putative conserved signature motifs and critical amino acid sites among *CNX* alignments are shown by different colored boxes (pink, red, blue, green, and deep blue) and are highlighted by shaded boxes (yellow and cyan).

The cloned full-length cDNA sequences of *TrCNX1* were deposited at the NCBI database under GenBank accession number: KU530113. Conserved domain signatures in TrCNX protein were analyzed through PFAM (http://pfam.xfam.org/search). The predicted TrCNX1 consisted of an N-terminal, conserved ER luminal of central and C-terminal transmembrane (TM) cytosol domains (Figure 1d), as found in an earlier study [22]. Based on the multiple sequence alignment of deduced amino acid sequences of *TrCNX1,* along with previously reported CNX proteins, *TrCNX1* was found to share common structural features of CNX proteins from other plant/animal species, including specific sites observed in a previous study [22], such as phosphorylation sites (PKC and CK2), N-myristoylation site, calrecticulin family signatures (1 and 2), and various other conserved signature motifs (Figure 1d, Figure 2).

**Figure 3:**
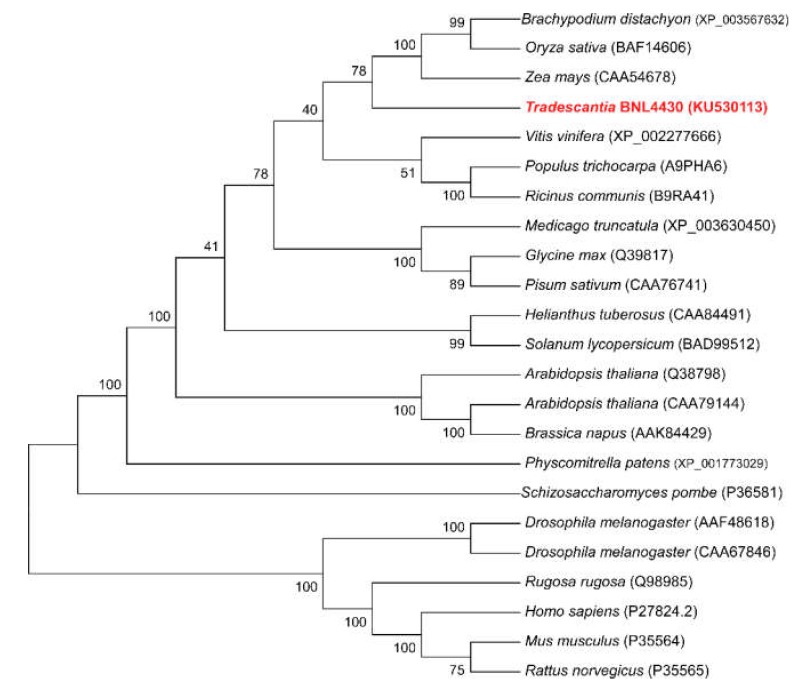
Phylogenetic tree of CNX based on the amino acid sequences from *Tradescantia* along with plant and non-plant species. A neighbor-joining tree was built using MEGA 4.02. A Dayhoff matrix-based method was used to estimate the branch support. The bootstrap values in branches were obtained by 1000 replications. Species name and accession numbers of CNX proteins represent the sources of CNX proteins in the figure.

To identify sequence homology and evolutionary relationships between *Tradescantia* and various plant and animal species based on the *CNX* gene, a phylogenetic neighbor-joining (NJ) tree was built using the deduced amino acid sequences of *CNX* genes. Phylogenetic analysis indicated that *TrCNX1* was closely related to monocotyledons, and clustered together with maize (CAA54678), Salniad *O*. *sativa* (BAF14606), and *Brachypodium distachyon* (XP_0003567632) on the same branch, and was distantly related to dicotyledons, such as *A*. *thaliana* (CAA79144) and *Solanum lycopersicum* (BAD99512) (Figure 3).

### 2.2. Differential Expression of TrCNX1 in Various Organs of *Tradescantia*

**Figure 4:**
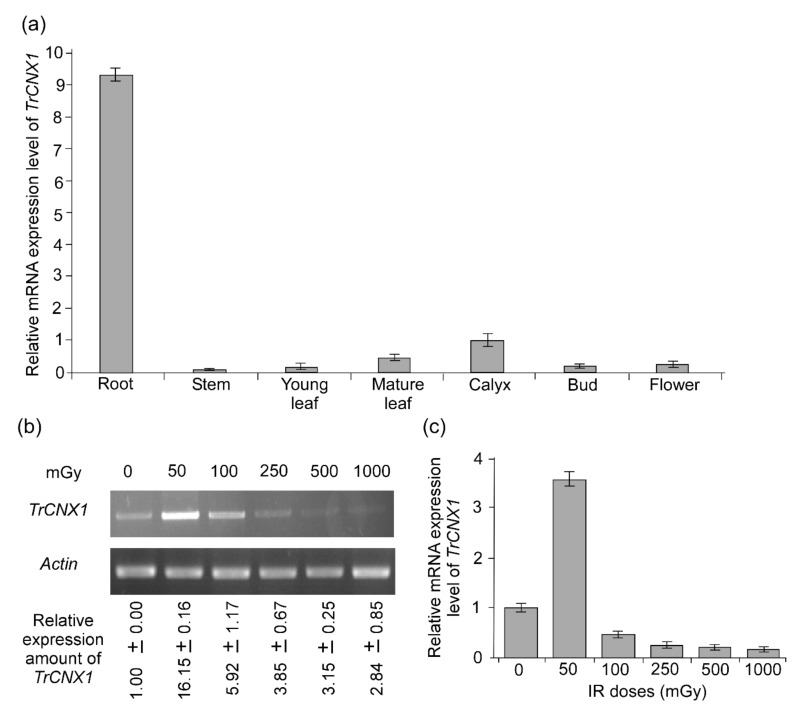
mRNA expression analysis of *TrCNX1* genes. The relative expression levels of *TrCNX1* genes were normalized to that of the *actin* reference gene. (**a**) Differential tissue expression of *TrCNX1* transcript in *Tradescantia*. *TrCNX1* transcript levels were investigated in seven different tissues via RT-qPCR. Transcriptional levels of calyx were set as control (the value of 1) to estimate expression in different organs. (**b**) RT-PCR validation of *TrCNX1* gene expression levels in response to various doses of gamma irradiation (Ɣ–IR) treatments (50, 250, and 500 mGy for 1 h, and 1000 mGy for 10 h). Lower panels indicate *Tradescantia* actin gene amplification. Values with ± standard error represent the relative amounts of calculated *TrCNX1* expression as described in the Materials and Methods section. (**c**) Relative mRNA expression levels of *TrCNX1* in response to various doses of Ɣ–IR t using RT-qPCR. Transcriptional levels with 0 mGy were set as the control (the value of 1) to determine the expression levels under other treatments.

*CNX*s are ubiquitously expressed in different plant tissues [21,22]. Therefore, the present study investigated the transcript accumulation of *TrCNX1* in seven different organs (mature and young leaves, stem, root, calyx, bud, and flowers) of *Tradescantia* using RT-qPCR (Figure 4a). The results showed that *TrCNX1* transcripts were ubiquitous in all the tissues analyzed. Furthermore, *TrCNX1* accumulated at the highest level in roots and the lowest level of in stems.

### 2.3. Accumulation of TrCNX1 Transcript in *Tradescantia* Flowers upon Ɣ-IR Stress

*CNX* is responsive to various abiotic stresses, including drought, cold, heat, salinity, and chemical/hormone (CaCl_2_, H_2_O_2_, abscisic acid, and zeatin) treatments [22,28]. In our transcriptome analysis, *TrCNX1* was also found to respond actively to Ɣ-IR stress, in particular to LDIR (50 mGy) in floral tissues. Therefore, to validate the mRNA expression levels of *TrCNX1* in floral tissues following exposure to different doses of Ɣ-IR stress (50, 100, 250, and 500 mGy for 1 h and 1000 mGy for 10 h), both RT-PCR and RT-qPCR analyses were performed. Since changes in the frequency of the blue-to-pink mutation in stamens and stamen hair cells (STC) of T-4430 usually occur 6–12 days after irradiation (DAI) [29], we collected floral samples at 12 DAI for the extraction of total RNA and subsequently performed RT-PCR and RT-qPCR (Figure 4).

The highest levels of *TrCNX1* mRNA were observed with 50 mGy and subsequently decreased along with increasing doses of Ɣ-IR (Figure 4b). Furthermore, the estimated rates of *TrCNX1* mRNA induction were approximately 16-fold higher than in the nontreated control at 50 mGy. This finding validates the observed transcript abundancy of *TrCNX1* at 50 mGy in our transcriptome study. This was further confirmed by RT-qPCR analysis, where *TrCNX1* was found to be highly upregulated under 50 mGy treatment (~3.5-fold) compared with the control (Figure 4c). In order to adapt under stress conditions, plants can sense changes in their surrounding atmosphere and can respond efficiently by altering their gene expression at molecular level, from which a protective response may follow that results in increases of stress tolerance. This phenomenon is referred to as protective feedback adaption, which usually shows to aid in the survival of plants in the stress environment. Therefore, the results of the RT-PCR and RT-qPCR analyses suggest that *Tradescantia* exerts protective feedback for adaptation to LD of Ɣ-IR stress by modulating the expression of stress-responsive genes at the transcriptional level.

### 2.4. Expression and Analysis of TrCNX1 Fusion Protein in Recombinant E. coli

**Figure 5:**
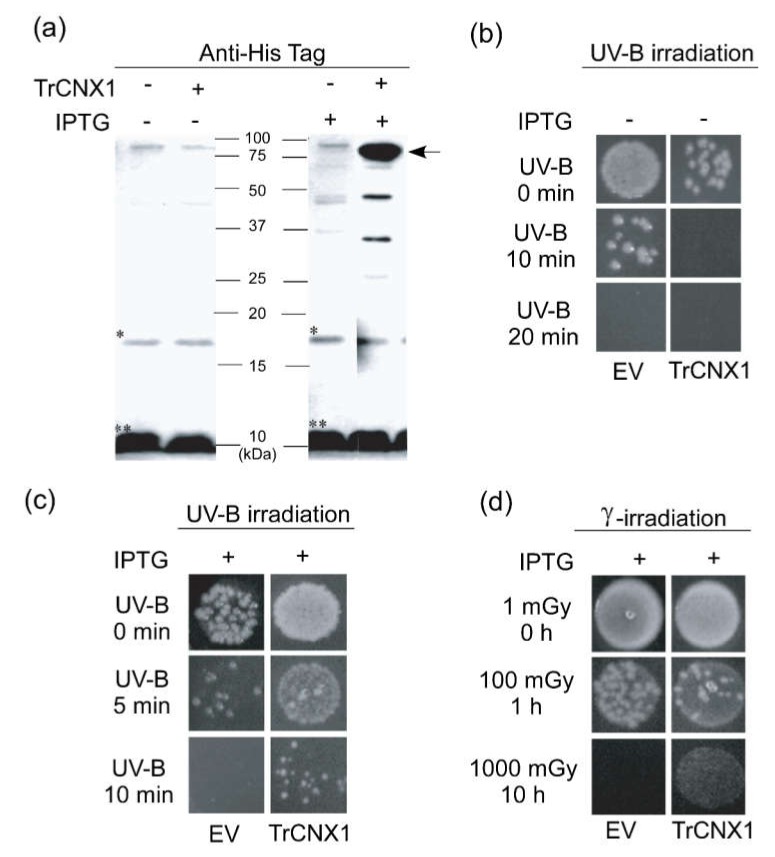
Analysis of recombinant TrCNX1 fusion proteins in *Escherichia coli* BL21(DE3) and growth of TrCNX1-expressing *E*. *coli* under irradiation stress (UV-B and Ɣ-IR). (**a**) Western blot analysis of overexpressed recombinant *TrCNX1* fusion proteins in *E*. *coli* with anti-His tag antibodies. The left and right panels denote the total proteins of *E*. *coli* cells without (–) and with (+) isopropyl-b-D-thiogalactoside (IPTG) induction, respectively. A protein ladder of 10–100 kDa is shown parallel to lanes indicating band sizes. Overexpressed *TrCNX-1 is* indicated by an arrow, while non-specific bands are shown by ‘* or **’. (**b**) Cell viability of *E*. *coli* transformants without IPTG induction in response to UV-B stress for 0, 10, and 20 min. Exponentially growing *E*. *coli* transformants of EV (harboring pET-28a empty vector (BL/EV) and TrCNX1 (harboring pET*-28a-TrCNX1* [BL/*TrCNX1*]) in liquid LB medium (without [–] IPTG supplement)) were serially diluted (1:10) and spotted on solid LB medium (with 50 μg/mL of kanamycin). Spotted plates were then subjected to UV-B stress. (**c**) Viability of (+) IPTG induced *E*. *coli* transformants under UV-B stress for different time points. (**d**) Viability of (+) IPTG-induced *E*. *coli* transformants in response to different doses of (100 and 1000 mGy) Ɣ-IR stress for different times (1 and 10 h).

To examine the functional role of *TrCNX1* in response to irradiation stresses, *TrCNX1* was heterologously overexpressed in *E. coli*, which is a convenient model for the functional study of foreign proteins. Kanamycin-resistant transgenic *E*. *coli* lines carrying recombinant BL/pET28a-*TrCNX1* (BL/*TrCNX1*) were confirmed by DNA sequencing. Overexpressed *TrCNX1* in *E*. *coli* was further validated by a Western blot analysis following SDS-PAGE. Recombinant pET28a-TrCNX1 was successfully overexpressed in *E*. *coli* after 1 h of IPTG (100 μM) treatment, while an *E*. *coli* cell line harboring BL/pET28a (EV) did not over-express signal regardless of IPTG treatment (Figure 5a). Furthermore, no proteins were over-expressed in *E*. *coli* cell lines harboring either BL/EV or BL/TrCNX1 without IPTG treatment, indicating that the heterologous expression of TrCNX1 was tightly controlled by IPTG treatment under the T7 promoter system in *E*. *coli*. The molecular mass of the proteins deduced from recombinant pET28a-TrCNX1 cells should be ~74 kDa. A specific band denoting an overexpressed protein in pET28a-TrCNX1 was detected at 75 kDa during Western blot (Figure 5a). These results demonstrated that recombinant TrCNX1 protein was efficiently expressed in *E*. *coli*.

### 2.5. Overexpressed TrCNX1 in *E. coli* Enhanced Resistance to Irradiation Stresses

To examine whether heterologously expressed TrCNX1 could increase bacterial cell viability in response to irradiation stress, BL/EV and BL/*TrCNX1* cells were used on a spotting assay. Prior to irradiation, the threshold rate (viability) of *E*. *coli* cells without IPTG induction under UV-B irradiation stress was determined, in which cells were diluted properly and spotted on plates, followed by exposure to UV-B stress for 10–20 min. Following UV-B exposure for 10 min, the number of cells in BL/EV and BL/*TrCNX1* were apparently lower and death in comparison to their corresponding control of 0 min, respectively (Figure 5b). The combined effect of UV-B and the integration of recombinant *TrCNX1* might have altered the whole-cell protein profile and expression of certain genes, which possibly resulted in death of cells in recombinant BL/*TrCNX1* when compared to the wild type of BL/EV under 10 min of UV-B stress without IPTG treatment. Meanwhile, no *E*. *coli* cells containing BL/EV or BL/*TrCNX1* survived under stress (Figure 5b), indicating that in the absence of *TrCNX1* gene expression, *E. coli* cells are susceptible to UV-B stress. Then, the survival of *E*. *coli* cells BL/EV and BL/*TrCNX1* was investigated under UV-B for 50—10 min and Ɣ-IR stresses for 0–10 hr following IPTG treatment.

Following UV-B stress for 5 min, the recombinant BL/*TrCNX1* cells displayed better growth and an increased number of colonies compared with the control BL/EV. After 10 min of treatment, all BL/EV cells were dead, while BL*/TrCNX1* cells displayed significant survival against UV-B stress (Figure 5c). This confirmed that the recombinant TrCNX1 protein was strongly induced by IPTG in BL*/TrCNX1* cells, which could augment the tolerance level in the bacterium. The results of the spot assay after Ɣ-IR stress revealed a similar survival growth pattern as observed in the UV-B stress experiment. Following 100 mGy treatment for 1 h, there were more colonies of BL/EV cells than BL/*TrCNX1* cells; however, a significant survival ratio was observed after HDIR with 1000 mGy, when there were 100-fold more BL/*TrCNX1* cells compared with BL/EV cells (Figure 5d). These results further suggest that the LD of Ɣ-IR (100 mGy) treatment under short-term (1 hr) exposure might act as a threshold, causing only minor damage to *E*. *coli* cells, thereby reducing the number of cells in BL/EV and BL/*TrCNX1*. At this threshold stage, the *E*. *coli* cells might have activated their molecular networks by modulating the up- and downregulation of certain gene expressions, including the *TrCNX1,* to prepare and overcome the radiation stress tolerance. Conversely, HDIR for a prolonged duration (10 hr) implies potentially lethal damage to *E*. *coli* cells, which eventually leads to the death of almost all cells in BL/EV when compared to BL/*TrCNX1* cells where overexpressed *TrCNX1* gene could have augmented the tolerance level. This result suggests that the *TrCNX1* gene increased the level of *E*. *coli* tolerance in response to Ɣ-IR stress. The present results indicate that *TrCNX1* is important for *E. coli* cell survival during irradiation stress, including UV-B and Ɣ-IR.

## 3. Discussion

In natural environments, plants respond to different kinds of abiotic and biotic stresses. These stress responses are usually regulated by specific signaling pathways and regulatory genes, which generally involve the accumulation of reactive oxygen species (ROS) in cells. This ROS accumulation induces stress-specific biomarkers at both the genome and proteome levels [30]. Identifying stress-specific biomarkers could be useful for crop improvements through breeding programs and for elucidating the stress response mechanisms in plants. Regarding abiotic stresses, certain types of irradiation, such as UV-B and Ɣ rays, also affect the normal growth and development of plants [3] and lead to ROS production, which can induce DNA damage. Recently, novel strategies have been suggested to measure the risk of radiation exposure and the long-term effects on living organisms using DNA damage as a potential biomarker [30,31]. Therefore, identifying IR-tolerant genes is important and is normally achievable by the transcriptome profiling of plants in response to IR stresses [16]. In our previous study, we obtained the flower transcriptome of T-4430 following exposure to Ɣ-IR (https://www.ncbi.nlm.nih.gov/bioproject/PRJNA612745). T-4430 is an ideal bioindicator and radiosensitive plant because of its mutation efficiency in terms of chromosome numbers and STC (blue to pink) when exposed to radiation [32,33]. Although the biological effects of IR on living organisms have been studied extensively, information on the effects of LDs of Ɣ-IR is still limited. Therefore, we subjected T-4430 plantlets to five different LDs of Ɣ-IR (50, 100, 250, and 500 mGy for 1 h and 1000 mGy for 10 h) in order to delineate the molecular responses and IR-tolerant genes in T-4430.

From our transcriptome assay, we identified several potential irradiation-responsive genes, which were strongly induced by LDIR, using *Tradescantia* BNL 3340 transcriptomic resources. Here, we functionally characterized an irradiation-responsive gene, called *calnexin-1,* from *Tradescantia* BNL 3340 (Figure 2 and Figure 3). Calnexin, also known as a molecular chaperone, is widely conserved among animals, plants, and fungi [22]. Calnexin has been reported to be an integral membrane protein, which plays a crucial role in the proper folding and secretion of newly synthesized nascent glycoproteins in the endoplasmic reticulum [34].

In this study, we isolated and cloned the full-length CDS of *Tradescantia* BNL 3340 *calnexin-1* (*TrCNX-1*) and further identified the intragenic regions (Figure 1). The deduced amino acid sequence alignments revealed that *TrCNX1* was highly homologous with plant *CNX*s (*AtCNX, BnCNX, SlCNX*, and *OsCNX*) and moderately homologous with that of yeast (*SpCNX*) (Figure 2). Casein kinase II phosphorylation sites (CK2) play crucial roles in regulating the functions of calnexin [35]. Thus, *TrCNX1* possessed eight conserved potential casein CK2 sites (one and seven sites in the N-terminal and central luminal domain, respectively) (Figure 2), as noted in other plants with *CNXs* in their coding regions [22]. Previous studies have suggested that these potential CK2 sites participate in signal transduction [36]. Moreover, four CK2 sites (D^277^, D^290^, E^333^, and T^440^) were highly conserved among plant and yeast CNXs (Figure 2).

CNX proteins of higher eukaryotes are reported to possess a high degree of sequence conservation, particularly in the highly conserved central domain (HCD), which contains KPEDWD repeats representing high-affinity calcium-binding sites (Ca^2+^) and lectin-oligosaccharide binding sites [19,37]. In addition, the HCD in calnexin has been implicated in the biosynthesis of cell wall components in yeast [37]. We noted that the HCD comprising Ca^2+^ and lectin–oligosaccharide binding sites was highly conserved in TrCNX1 (residues 331–339 and 343–354). Ca^2+^ sites are essential for the lectin-like behavior of CNXs [37]. In animals, lectin–oligosaccharide binding sites help CNX to interact with glycosylated and nonglycosylated proteins in the ER [38]. However, the interactions of plant CNXs and other substrate proteins are presently unclear. Furthermore, the primary sequence structure of TrCNX and other plant CNXs is highly homologous with that of yeast CNXs (DmCNX and HSCNX) when comparing the amino-terminal domain (residues 27–54 and 65–77) and carboxyl-terminal (residues 577–582 and 591–596) (cytosolic domain), which are important for the function of wild-type SpCNX1 [37].

Phylogenetic analysis of various CNX proteins revealed that TrCNX1 was closely associated with monocotyledon CNXs (maize, rice, and Brachypodium) (Figure 3). Three copies of CNX exist in Arabidopsis [17], while only one copy is found in *Tradescantia,* as observed in rice [22], showing the possible functional divergence between dicot and monocot plant systems. In yeast (S*accharomyces pombe*), a single copy of CNX acts as the sole protein-folding machinery for survival of cells [37]. Moreover, CNXs localize to the ER, indicating that they have major roles in the physiological status of cells. CNX mRNA accumulates ubiquitously in a wide range of tissues [21,22]. Here, we found that *TrCNX1* was present in all tissues (leaves of mature and young plants, stems, roots, and flower parts, including the bud, calyx, and petal) (Figure 4a), which corresponds to previous findings that CNXs might be involved in the general growth and development of plants [22]. Furthermore, *TrCNX1* was found to be highly expressed in roots, suggesting a possible physiological role in root development [28]. Ca^2+^ ions are important secondary messengers in signaling pathways involved in the adaptive responses of plants to various adverse environmental conditions, such as drought, salinity, temperature, and pathogen attack [39].

We investigated the accumulation of *TrCNX1* mRNA in response to Ɣ-IR stress using RT- and RT-qPCR (Figure 4b,c). Both analyses revealed that *TrCNX1* was strongly induced in flowers upon different doses of Ɣ-IR stress; notably, high accumulation of *TrCNX1* transcripts was observed in response to 50 mGy (Figure 4b,c) compared with control or higher doses (<100–1000 mGy). This high accumulation of *TrCNX1* under LD Ɣ-IR stress suggests that it is involved in LDIR stress tolerance in plants. In contrast, higher doses (<100–1000 mGy) had no significant impact on the mRNA accumulation of *TrCNX1*. This suggests that LDIR might have a higher impact on living organisms in a distinct way to HD Ɣ-IR stress. Supporting this hypothesis, previous studies have already suggested that LDIR has more genetic consequences than the HDIR in plant and animal cells [9]. This suggests that 0 mGy may be the threshold for *TrCNX1* expression, and a complex network of different stress-response pathways, including elevated expression of ER chaperone genes, could be triggered by very high levels LDIR stress (50 mGy), while relatively high levels of LDIR (100–1000 mGy), such as >50 mGy, induce damage to ER machinery by downregulating the *TrCNX1* (Figure 4c).

The ER is sensitive to adverse environmental conditions. ER stress is induced by the unfolded-protein response (UPR), which triggers the transcriptional upregulation of genes encoding ER-resident chaperones such as *binding protein* (Bip), *protein disulfide isomerases* (PDI), *calnexin* and *calreticulin* [27,40], and other regulatory systems to maintain the optimum atmosphere within the ER under stress conditions. Expression of *TrCNX1* (at 50 mGy) is increased in response to ER stress resulting from abnormal levels of unfolded proteins following Ɣ-IR exposure. This might have implications for the regulation of unfolded proteins within the ER machinery under stress conditions. To support this, the present study heterologously overexpressed the TrCNX1 in *E. coli*. The results indicate that overexpression of TrCNX1 protein significantly enhanced the level of *E. coli* tolerance to radiation stress induced by UV-B and Ɣ-IR. Transformation of the *TrCNX1* gene in *E. coli* cells increased endogenous levels of TrCNX1 proteins in the presence of IPTG (Figure 5a). During the spot assay, BL/TrCNX1 recombinant *E. coli* cells exhibited a higher number of colonies under radiation stresses compared with control BL/EV (Figure 5c,d). This confirms that *TrCNX1* has a protective role against irradiation stress. The functions of plant CNX proteins have not been well characterized in previous studies. Previous reports have suggested that CNX acts as chaperones in ER and enhances drought stress tolerance in plants. Overexpression of *OsCNX* was found to increase drought tolerance in tobacco [22]. 

In conclusion, this study characterized the novel irradiation-tolerance gene *TrCNX1*, which encodes a 61.4 kDa protein. Overexpression of *TrCNX1* in an *E*. *coli* heterologous system confirmed its protective role against UV-B and Ɣ-IR stress, demonstrating that it could function in molecular networks of radiation tolerance in the clone T4430. Further work is required to explore its mechanisms of protection against irradiation stresses in transgenic plants.

## 4. Materials and Methods

### 4.1. Plant Materials and Radiation Treatment

*Tradescantia* clone BNL 4430 (T-4430) (2n = 12) is an interspecific hybrid between *T. hirsuiflora* and *T. subacaulis* (Sparrow, 1974), which was obtained from the Korea Atomic Energy Research Institute (KAERI), South Korea. All T-4430 plants were planted in 20 cm plastic pots with a potting mixture (1 soil: 1 sand: 1 Perlite) and maintained in a greenhouse at 25 and 20 °C under 16 and 8 h light and dark conditions, respectively. Relative humidity of 70%–80% was maintained, and all plants were irrigated at a 2 day interval. Eight-weeks-old mature plants were subjected to Ɣ-IR stress using a gamma-phytotron facility equipped with a ^60^Co source (KAERI). All tissue samples collected from the experimental plants were immediately frozen in liquid nitrogen and stored at –80 °C for further analysis.

### 4.2. Transcriptome Resource, in Silico Identification, and Sequence Analysis

A de novo-assembled EST library was recently developed in our laboratory from T-4430 flowers following exposure to Ɣ-IR stress (unpublished raw data, available under accession number PRJNA612745 at NCBI: http://www.ncbi.nlm.nih.gov/bioproject/PRJNA612745). These transcriptomic resources were used in the present study to identify sequence information of several radiation-responsive genes, including *Tr-CNX-1*. To compare the *Calnexin-1* nucleotide and deduced amino acid sequences of *Tradescantia* with those of other species, a multiple sequence alignment was performed using BioEdit 7.0 (Therapeutic, Carlsbad, CA, USA). Evolutionary relationships among members of the *CNX* gene family from different plant species were analyzed by constructing a phylogenetic neighbor-joining (NJ) tree using MEGA5.1 software [41].

### 4.3. mRNA Isolation, c-DNA Cloning, RT-PCR, and RT-qPCR Analysis

Total RNA was isolated from various tissues (root, stem, young leaf, mature leaf, calyx, bud, and flower), including Ɣ-IR-treated floral tissues of *Tradescantia* using the GeneAll Hybrid RNA purification kit (GeneAll Biotechnology, Daejeon, Korea) according to the manufacturer’s protocol. Primers were designed to amplify transcripts based on the sequence information of *TrCNX1* (KU530110) and *TrActin-7* (KX173444) and are listed in Table S1. cDNA was synthesized from 2 μg of total RNA using TaKaRa RNA PCR Kit (AMV) Ver.3.0 (TAKARA Bio, Kyoto, Japan), according to manufacturer’s instructions. PCR was carried out with the following parameters: 30 °C for 10 min, 42 °C for 20 min, and 95 °C for 5 min. mRNA expression of *TrCNX-1* was investigated by both RT-PCR and RT-qPCR. RT-PCR was performed in 50 µL reaction mixtures containing 100 ng of reverse-transcribed cDNA as a template, 1× PCR buffer, 1 mM dNTP, 1.25 units of EX-Taq (TAKARA Bio, Kyoto, Japan), and 0.2 μM of gene-specific forward and reverse primers. RT-PCR conditions were 95 °C for 2 min initially, followed by 25 cycles of 94 °C for 30 s, 50 °C for 30 s, and 72 °C for 1 m 50 s. During RT-PCR, bands for *TrCNX1* were quantified using ImageJ 1.46 software (NIH, Bethesda, MD, USA). RT-PCR data were expressed as the relative amounts of *TrCNX1,* normalized to expression of actin genes with a mean ± standard error of three independent experiments. For RT-qPCR, a 20 μL reaction volume containing 2 μL of reverse-transcribed cDNA, 2× quantispeed SYBR^®^ Green mix (PhileKorea, Daejeon, Korea), and 0.5 μM of gene-specific forward and reverse primers were used to calculate the mRNA expression level of *TrCNX* using the Eco^TM^ Real Time PCR System (Illumina, Seoul, Republic of Korea). The RT-qPCR was performed according to our previous study in Tradescantia [42]. Using three biological replicates for each gene in the PCR, the relative expression levels of *TrCNX-1* were normalized according to that of the reference gene, *actin-7,* using the 2^-ΔΔCT^ method [43].

### 4.4. Cloning of TrCNX into the pET28(a) Expression Vector and Recombinant Protein Expression

Cloned cDNA sequences of the *TrCNX-1* gene, without any stop codons, were inserted into the overexpression vector pET-28a (+) using primers with flanking *NcoI* restriction sites in the forward primer and *XhoI* in the reverse primer (Table S1) to produce the recombinant pET-28a-*TrCNX-1* expression vector. The pET-28a-*TrCNX-1* plasmid was transformed into BL21(DE3). *E. coli* transformants harboring pET-28a served as a control.

### 4.5. Spot Culture Assay to Analyze the Effects of UV-B and Ɣ-Radiation Stress

*E. coli* transformants harboring pET-28a (control) and pET-*TrCNX1* were grown overnight at 37 °C in liquid LB medium. Then, the cultures were diluted in fresh LB medium at a 1:40 ratio and grown at 37 °C. When the cell density reached 0.2 at OD600, 100 μM of isopropyl-b-D-thiogalactoside (IPTG) was added, and the cells were again grown for 1 h or until a density of 0.5 was reached at OD600. Then, the cells were spotted onto solid LB-medium (50 μg/mL kanamycin, 100 μM of IPTG) at serial dilutions of 1:10. The spotted *E. coli* plates were subjected to UV-B (non-IR) and Ɣ-radiation (IR) stress for different times. UV-B (non-IR) treatment was carried out using a UV transilluminator (DAIHAN Scientific, Korea), while Ɣ-radiation (IR) treatment was carried out using a phytotron machine with a 60Co radiation source, available at KAERI, Jeongeup. All irradiated plates were incubated at 37 °C for 24 h, and then cell viability was analyzed by employing the plate count method to observe the number of colonies and growth pattern. Triplicate experiments were performed independently.

## Supplementary Materials

The following are available online at https://www.mdpi.com/2223-7747/9/3/387/s1, Table S1: List of primers designed and used in this study.
