# Supplementary material for: Molecular Characterization and Identification of Calnexin 1 As a Radiation Biomarker from Tradescantia BNL4430
Source: Plants (Basel). 2020 Mar 20;9(3):387. doi: 10.3390/plants9030387 (PMC7154805; doi:10.3390/plants9030387)
Supplement: Supplementary file 1 [file plants-09-00387-s001.pdf]

Table S1. List of primers designed and used in this study.

Primer name	Primer sequence (5′→3′)	Relevance	Annealing (Tm °C)	PCR Length (bp)
G-TrCNX-F<br>G-TrCNX-R	ATGAGACAACGGCAACTATTTTCCG<br>TCAAAATCTACGCCCTCCTCC	gDNA-PCR	62	3069
TrCNX-F<br>TrCNX-R	ATGACGATCGCGCCAAAGATC<br>TCAAAATCTACGCCCTCCTC	cDNA-PCR	61	1850
RT-CNX-F<br>RT-CNX-R	TGGCAGCATACAAGTCCAAG<br>GCTCGTTCCCTTCCTTCTCT	RT-PCR	59	240
q-CNX-F<br>q-CNX-R	GAGAAGCAGAAGGCTGAAGAG<br>GGAACATCAGCTACCTTGTAGAG	RT-qPCR	60	106
RT-Actin7-F<br>RT-Actin7-R	GACCGATTCCCTGATGAAGA<br>ATGATGGCTGGAACAGAACC	RT-PCR	58	242
qRT-Actin7-F<br>qRT-Actin7-R	CTGATAGGATGAGCAAGGAGA<br>ATAGACCCTCCAATCCAGACA	RT-qPCR	60	105
TrCNX1-NcoI-F<br>TrCNX1-NcoI-R	**CCATGG**ATGACGATCGCGCCAAAGAT<br>**CTCGAG**TCAAAATCTACGCCCTCCTC	Subcloning of *TrCNX1*	60	1629
